# Impact of tetrachloroethylene-contaminated drinking water on the risk of breast cancer: Using a dose model to assess exposure in a case-control study

**DOI:** 10.1186/1476-069X-4-3

**Published:** 2005-02-25

**Authors:** Verónica Vieira, Ann Aschengrau, David Ozonoff

**Affiliations:** 1 Department of Environmental Health, Boston University School of Public Health, 715 Albany Street, Boston, MA 02118, USA; 2 Department of Epidemiology, Boston University School of Public Health, 715 Albany Street, Boston, MA 02118, USA

## Abstract

**Background:**

A population-based case-control study was undertaken in 1997 to investigate the association between tetrachloroethylene (PCE) exposure from public drinking water and breast cancer among permanent residents of the Cape Cod region of Massachusetts. PCE, a volatile organic chemical, leached from the vinyl lining of certain water distribution pipes into drinking water from the late 1960s through the early 1980s. The measure of exposure in the original study, referred to as the relative delivered dose (RDD), was based on an amount of PCE in the tap water entering the home and estimated with a mathematical model that involved only characteristics of the distribution system.

**Methods:**

In the current analysis, we constructed a personal delivered dose (PDD) model that included personal information on tap water consumption and bathing habits so that inhalation, ingestion, and dermal absorption were also considered. We reanalyzed the association between PCE and breast cancer and compared the results to the original RDD analysis of subjects with complete data.

**Results:**

The PDD model produced higher adjusted odds ratios than the RDD model for exposures > 50^th ^and >75^th ^percentile when shorter latency periods were considered, and for exposures < 50^th ^and >90^th ^percentile when longer latency periods were considered. Overall, however, the results from the PDD analysis did not differ greatly from the RDD analysis.

**Conclusion:**

The inputs that most heavily influenced the PDD model were initial water concentration and duration of exposure. These variables were also included in the RDD model. In this study population, personal factors like bath and shower temperature, bathing frequencies and durations, and water consumption did not differ greatly among subjects, so including this information in the model did not significantly change subjects' exposure classification.

## Background

In 1988, an unusually high incidence of cancer in the Cape Cod region of Massachusetts prompted a series of epidemiological studies to investigate possible environmental risk factors associated with the region, including tetrachloroethylene-contaminated drinking water [1-7]. Tetrachloroethylene (or perchloroethylene, PCE) entered the drinking water when it leached from vinyl liners of water distribution pipes introduced in the late 1960s. When the contamination was discovered, the Massachusetts Department of Environmental Protection began flushing and bleeding the pipes in 1980. At that time, the suggested limit set by the Environmental Protection Agency (EPA) was 40 ppb [8], but has since been lowered to a mandatory Maximum Contaminant Level (MCL) of 5 ppb.

A population-based case-control study was undertaken to investigate the association between tetrachloroethylene exposure from public drinking water and breast cancer [5]. The study defined exposure using a cumulative measure Webler and Brown termed the relative delivered dose (RDD) [9]. Calculations for the RDD use the rate at which PCE leached from the pipe liner, the surface area of the interior of the pipe, and the upstream load. The RDD is relative to the total delivered mass of PCE entering each residence over time, but the constants and variables assumed to be constant were dropped from the analysis. While this allowed for grouping of the population into exposure categories, the RDD value computed is not an actual water concentration. Refer to Webler and Brown for a detailed description of the RDD model [9].

Because PCE is a volatile organic chemical that readily escapes from water into air, the amount of PCE inhaled during showers and baths, as well as the amount ingested and dermally absorbed, was relevant. The RDD measure does not consider these exposure pathways, which could potentially result in bias from exposure misclassification. Using personal exposure factors such as tap water consumption and bathing habits, we constructed a dose model to quantify the relative amount of PCE taken in by each subject, which we refer to as the personal delivered dose (PDD). The dose values calculated by the PDD model were subsequently used to measure the strength of the association between PCE exposure and the risk of breast cancer. The objective was to see if additional information contained in individual survey data affected associations between breast cancer and PCE exposure.

## Methods

### Study Population

The population-based case-control study was designed to evaluate the association between breast cancer and tetrachloroethylene (PCE) exposure from public drinking water [5]. During the period 1987–1993, the Massachusetts Cancer Registry recorded 672 incident cases of female breast cancer among permanent residents of the Massachusetts towns Barnstable, Bourne, Brewster, Chatham, Falmouth, Mashpee, Provincetown, and Sandwich, where pipes with PCE-containing vinyl liners had been installed.

Female controls were chosen to represent the underlying population that gave rise to the cases. Selection criteria required controls to be permanent residents of the same towns during 1987–1993. Controls were frequency matched to cases on age and vital status. Because many of the cases were elderly or deceased, three different sources of controls were used: (1) random digit dialing identified living controls less than 65 years of age; (2) Centers for Medicare and Medicaid Services, formerly the Health Care Financing Administration, identified the living controls 65 years of age or older; and (3) death certificates identified controls who had died from 1987 onward. The resulting 616 controls provide an estimate of the exposure distribution in the underlying population.

Subjects or their next-of-kin completed extensive interviews, which provided information on demographics (e.g., age, sex, marital status, education), a 40-year residential history, and potential confounders (e.g., age, family history of breast cancer, age of first live or still birth, oral contraceptive use). Next-of-kin served as proxies for cases and controls who were deceased or too ill to participate in the interview. "Index years" were randomly assigned to controls to achieve a distribution similar to that of cases' diagnosis years and only exposures before the diagnosis year (for cases) and index year (for controls) were counted. The analysis considered a range of latent periods: 0, 5, 7, 9, 11, 13, 15, 17, and 19 years. For a detailed description of the methods, see Aschengrau et al. [5].

### Dose Model

If individual behavior in water use is an important element in a person's exposure, using the relative delivered dose (RDD) could bias the results. The RDD quantifies the amount of PCE in the drinking water, but does not consider exposure from inhalation, dermal absorption, and ingestion. PCE is a volatile organic compound and daily indoor inhalation exposure to contaminated water from showering can be up to six times greater than exposure from ingestion [10]. To further quantify dose and reduce exposure misclassification, a number of personal factors (e.g., bottled water consumption, duration and frequency of showers and baths) were considered.

Non-proxy cases and controls were interviewed about many of these factors: the number of glasses of tap water consumed per day, including drinks made with tap water, such as coffee or lemonade; the use of bottled water; and the temperature, frequency, and duration of showers and baths. Information on a subject's physical characteristics, such as height and usual weight, was also obtained. Certain model parameters not provided by the questionnaire were obtained from the current scientific literature (e.g., inhalation rate, water flow rate, air exchange rate).

We used this information to construct a personal delivered dose (PDD) model that considered three exposure routes: inhalation, dermal absorption, and ingestion. The RDD value was converted into an annual concentration and used as the initial water concentration for the PDD model (mg/L). The amount of PCE contributed by inhalation is a function of the temperature, frequency and duration of baths and showers, and the concentration of PCE in the bathtub/shower stall air. To determine the amount of PCE that volatilized from the water, the two-resistance theory was applied to temperature dependent physical and chemical properties of PCE [11]. The dermal absorption component of the model estimated each subject's surface area (from her height and weight) and determined the amount of PCE absorbed during baths and showers using Fick's first law [12]. The amount of PCE that a subject ingested was dependent on the volume of tap water consumed. By summing the total amount of PCE from the three exposure routes over all exposed residences, we arrived at a personal delivered dose (PDD) for each subject. A detailed description of the dose model is provided in Additional file 1: Dose Model Appendix.

### Data Analysis

Questions regarding tap water use and bathing habits were not asked in proxy interviews so the PDD analysis was restricted to non-proxy subjects (n = 885, Table 1). To accurately compare results from the RDD and PDD analyses, we first recalculated associations using the original RDD exposure measure for only the non-proxy subjects. Women with cumulative RDD exposures were compared with never-exposed women. Never-exposed women did not live downstream of vinyl-lined pipes.

**Table 1:** Number of subjects by proxy/non-proxy, PCE-exposed/unexposed, and case/control status.

	**Non-Proxy Subjects**	**Proxy Subjects**	**Total Subjects**
**PCE-exposed**	**189**	**102**	**291**
Cases	101	54	155
Controls	88	48	136
**Unexposed**	**696**	**301**	**997**
Cases	360	157	517
Controls	336	144	480
**Total**	**885**	**403**	**1288**
Cases	461	211	672
Controls	424	192	616

We defined a series of four exposure levels based on the exposure distribution of exposed controls. The lowest exposure level included all exposed subjects with RDD values less than or equal to the 50^th ^percentile. The remaining exposure levels were nested and included all RDD values greater than the 50^th ^percentile, greater than the 75^th ^percentile, and greater than the 90^th ^percentile. Therefore, a subject exposed at > 90^th ^percentile was also considered exposed at > 75^th ^and >50^th ^percentiles. We chose to nest exposure categories because there were too few subjects for mutually exclusive categories. There are no previous studies comparing nested exposure categories to mutually exclusive exposure categories.

Exposure groups were further categorized for latent periods that ranged from 0 to 19 years. Each exposure level was treated as a binary variable in separate multiple logistic regression models. Odds ratios (ORs) were calculated for each exposure level relative to never-exposed cases (n = 360) and controls (n = 336). The adjusted analysis controlled for a group of core confounders: age at diagnosis or index year, family history of breast cancer, personal history of breast cancer (before current diagnosis or index year), age at first live birth or stillbirth, and occupational exposure to PCE. These factors were chosen as confounders *a priori *based on the current scientific literature. Additional potential confounders were added to the logistic regression models along with the core confounders, including history of benign breast disease; past use of diethylstilbestrol, oral contraceptives, and menopausal hormones; cigarette smoking history; alcohol drinking history; history of ionizing radiation treatment; quetlet index (measure of obesity); race; marital status; religion; education level; and physical activity level. None of these additional variables changed the adjusted estimates by more than 10%, and so the final models included only the core confounders. Adjusted analyses were not performed if there were fewer than three exposed cases and three exposed controls in an exposure level [5]. We calculated 95% confidence intervals (CIs) for the adjusted ORs using maximum likelihood estimates of the standard errors [13].

We then repeated the crude and adjusted analyses using each subject's personal delivered dose (PDD) as an exposure measure. The PDD distributions of the exposed controls were used to define the same four exposure levels: less than or equal to the 50^th ^percentile, greater than the 50^th ^percentile, greater than the 75^th ^percentile, and greater than the 90^th ^percentile. The referent category remained never exposed cases and controls.

We also conducted a goodness-of-fit analysis to compare the RDD and PDD exposure measures and to determine which model performed better [14]. We compared the deviance of the models at different exposure levels and latencies. Lastly, we performed a nonparametric rank test to determine if the ranks of the subjects' PDD exposures differed significantly from the ranks of their RDD exposures.

## Results

### RDD analysis

We were interested in comparing the results of the Aschengrau et al. RDD analysis using all subjects to the restricted analysis performed on only non-proxy subjects. The distributions of core confounders were similar among non-proxy and all subjects, except non-proxy subjects were younger than all subjects (Table 2). The number of exposed subjects was reduced by 35% when proxies were removed (from 291 to 189) when no latency was considered. The number of unexposed subjects used as a common reference group for all analyses was reduced by 30% (from 997 to 696). The median, 75^th ^percentile, and 90^th ^percentile RDD values for the non-proxy exposed controls were similar to the values for the exposed controls among all subjects (Table 2). We compared analyses for ever vs. never PCE-exposed and found that the odds ratios were similar for the non-proxy subjects and all subjects (Table 3) [5].

**Table 2:** Distribution of selected confounders of breast cancer subjects (%) and RDDs of PCE-exposed controls.

Characteristic	**Non-Proxy **Cases (n = 461)	**Non-Proxy **Controls (N = 424)	**All **Cases (n = 672)	**All **Controls (N = 616)
Age at diagnosis or index years				
1–49 years	19.7 (91)	20.6 (87)	16.5 (111)	16.7 (103)
50–59 years	13.7 (63)	17.0 (72)	12.2 (82)	13.6 (84)
60–69 years	33.0 (152)	31.1 (132)	31.5 (211)	29.9 (184)
70–79 years	28.8 (133)	25.2 (107)	28.4 (191)	26.0 (160)
80+ years	4.8 (22)	6.1 (26)	11.4 (77)	13.8 (85)
Age at first birth or stillbirth				
< 30 years	60.6 (279)	65.7 (278)	61.0 (410)	66.8 (411)
30+ years	13.8 (64)	12.4 (53)	14.5 (97)	12.8 (79)
Nulliparous	25.6 (118)	21.9 (93)	24.5 (165)	20.4 (126)
Prior breast cancer	5.6 (26)	3.3 (14)	5.4 (36)	4.8 (30)
Family history of breast cancer	24.3 (112)	15.8 (67)	25.6 (172)	15.5 (95)
Occupational exposure to PCE	16.3 (75)	16.0 (68)	15.5 (104)	14.8 (91)

RDD Exposure (for no latency)				
Minimum	---	0.001	---	0.001
Maximum	---	206.9	---	243.8
Median	---	2.9	---	2.5
75^th ^percentile	---	11.9	---	12.1
90^th ^percentile	---	31.0	---	29.2

**Table 3:** Tetrachloroethylene exposure history of breast cancer subjects, adjusted^a ^odds ratios, and 95% confidence intervals.

	Latency period, years	Exposed Cases	Exposed Controls	Adjusted ORs (95 % CI)
0	Non-proxy Subjects	101	88	1.1 (0.8–1.5)
	All Subjects	155	136	1.1 (0.8–1.4)
5	Non-proxy Subjects	87	69	1.2 (0.9–1.8)
	All Subjects	129	107	1.2 (0.9–1.6)
7	Non-proxy Subjects	71	61	1.1 (0.8–1.6)
	All Subjects	111	96	1.1 (0.8–1.5)
9	Non-proxy Subjects	63	57	1.1 (0.7–1.6)
	All Subjects	97	85	1.1 (0.8–1.5)
11	Non-proxy Subjects	49	43	1.1 (0.6–1.7)
	All Subjects	79	65	1.2 (0.8–1.7)
13	Non-proxy Subjects	43	32	1.3 (0.7–2.1)
	All Subjects	61	45	1.3 (0.9–2.0)
15	Non-proxy Subjects	30	21	1.4 (0.7–2.6)
	All Subjects	44	31	1.4 (0.9–2.3)
17	Non-proxy Subjects	15	15	1.0 (0.4–2.2)
	All Subjects	21	21	1.0 (0.6–2.0)
19	Non-proxy Subjects	6	6	1.1 (0.3–3.5)
	All Subjects	9	9	1.1 (0.4–2.9)

^a ^The OR was calculated relative to never-exposed cases (n = 360 for **Non-Proxy**, n = 517 for **ALL**) and controls (n = 336 for **Non-Proxy**, n = 480 for **All**). Controlled for age at diagnosis or index year, family history of breast cancer, personal history of breast cancer (before current diagnosis or index year), age at first live-birth or still birth, occupational exposure to PCE, and vital status at interview (for **All **analysis only).

### PDD analysis

The distribution of cumulative RDD and PDD values ranged by five orders of magnitude, equivalent to a range from micrograms to hundreds of milligrams. Of the 189 exposed subjects in the no latency analysis, the personal delivered dose model changed the exposure categories of 39 subjects. However, the result from a non-parametric signed rank test indicates that the subjects' RDD ranks and PDDs rank are not significantly different (p = 0.81).

In general, odds ratios from the PDD analysis were slightly higher than the RDD analysis for exposure levels above the 50^th ^and 75^th ^percentiles at shorter latency periods (see Additional file 2: Table 4). At longer latencies, the ORs for the lowest and highest exposure groups in the PDD analysis were slightly higher than the RDD analysis, but small numbers of exposed subjects limited the adjusted analyses. The odds ratios for breast cancer increased with increased latency and higher exposure categories, although the odds ratios were not statistically significant. The confidence intervals were generally the same width for both the RDD and PDD analyses; they included the null value in both analyses, and grew wider as the exposure level and latency increased. Overall, the results from the PDD analysis did not differ greatly from the RDD analysis and any differences were well within the variation present in the RDD data, which formed the "input" to the PDD analysis.

The best fitting model is often but not always the one that produces the higher odds ratio [14]. The deviance measure of goodness-of-fit was smaller for the PDD than the RDD model at shorter latencies and lower exposure levels and larger at longer latencies and higher exposure levels. However, the close agreement between the goodness-of-fit measures suggests that there is little difference between the two models (see Additional file 3: Table 5). Further evidence of this is provided by the results of the nonparametric rank test, which indicated the two exposure rankings were not statistically different.

## Discussion

The dose model was constructed to reduce nondifferential exposure misclassification due to variations in personal behavior. In the RDD analysis, exposure was based solely on subjects' RDD values and did not take into consideration factors such as bathing habits and bottled water consumption. Nondifferential exposure misclassification should bias results towards the null when the exposure is dichotomous. Based on this reasoning, we expected the moderate elevations in risk observed in the RDD analysis by Aschengrau et al. [5] to increase further in the current PDD analysis. The results show that, in general, this was not the case.

Overall, the risks calculated from the PDD analysis differed only slightly from the RDD analysis, if at all. The fact that the PDD model did not increase the odds ratios may be due to a number of reasons. A possible explanation is that no association exists between exposure to PCE and breast cancer, but there is a fairly large body of literature now that supports a carcinogenic effect for PCE in humans. The biologic rationale for a breast cancer effect stems from a hypothesis described by Labreche and Goldberg that organic solvents such as PCE may act either directly as genotoxic agents or indirectly through their metabolites to increase the risk of breast cancer [15].

More likely, the impact of variations in personal habits was small in comparison to variations in characteristics of the drinking water distribution system, or the questionnaire information did not accurately account for individual variations. Errors in estimating the RDD values used in the dose model may explain why the model made little difference in determining risk. Improper assumptions or incorrect input variables in the Webler-Brown model led to errors in the RDD values [5]. The resulting exposure misclassification would not be corrected using the dose model. As a result, the dose model would still be biased.

Furthermore, both RDD and PDD are measures of cumulative exposure, where exposure was summed over a subject's residences on Cape Cod. One subject may have been exposed at a high intensity for two or more short residency durations while another subject with the same exposure value may have been exposed at a low intensity for one long residency duration. The exposure pattern can influence cancer risk if, for example, a threshold intensity of PCE must be reached in order to cause breast cancer or if breast cancer induction requires prolonged continuous exposure [16].

Another limitation of the analysis was the restriction to subjects with non-proxy interviews, which reduced the sample size by 31%. When all subjects were included in the RDD analysis, small to moderate increases were observed among women whose exposure level was greater than the 90^th ^percentile [5]. When only non-proxy subjects were included, we no longer observed moderate increases. This difference may be due to the fact that the maximum RDD value was higher for all subjects than for non-proxies. Therefore, the use of only non-proxy subjects may not accurately reflect population risk. Imputing values for proxy subjects is a possible option for future analyses.

Faulty recall in the behavioral data is another possible reason why the PDD model did not strengthen the association between breast cancer and PCE. Subjects were asked to remember details about bathing habits and drinking water that occurred up to forty years before the interview. As a result, the exposure data obtained at interview may not be accurate.

The inputs that most heavily influenced the PDD model were initial water concentration and duration of exposure. These variables were also included in the RDD model. In this study population, personal factors like bath and shower temperature, bathing frequencies and durations, and water consumption did not differ greatly among subjects. Therefore, including these characteristics in the PDD model did not significantly improve the exposure measure or change which subjects were considered exposed and to what level they were exposed.

## Conclusion

In an attempt to characterize PCE exposure more precisely, we constructed a dose model that considered exposure from inhalation, ingestion, and dermal absorption. The model incorporated personal information on tap water use and bathing habits obtained from study interviews. The dose values calculated by the model were subsequently used to measure the strength of the association between PCE exposure and the risk of breast cancer.

Although our results from the PDD analysis did not differ greatly from the RDD analysis, it remains important to assess exposure as accurately as practical in an epidemiological investigation. Many factors such as tap water use and bathing habits could be considered when determining exposure to volatile chemicals in domestic water supplies, but our analysis suggests that the use of such ancillary data does not always result in an improvement in exposure accuracy if the ancillary data are inaccurate or if they have little effect on an individual's exposure level.

## Abbreviations

PCE, Tetrachloroethylene; RDD, Relative Delivered Dose; PDD, Personal Delivered Dose; EPA, Environmental Protection Agency; MCL, Maximum Contaminant Level; ORs, Odds Ratios; CIs, Confidence Intervals

## Competing Interests

The author(s) declare they have no competing interests.

## Authors' Contributions

VV created the dose model, conducted the statistical analyses, and drafted the manuscript. AA provided the data and assisted in epidemiologic analysis and editing. DO participated in the design of the study and the editing of the manuscript. All authors read and approved the final manuscript.

## Supplementary Material

Additional File 1 This document describes the dose model in more detail. Click here for file

Additional File 2 This document provides a table of adjusted odds ratios for breast cancer by tetrachloroethylene exposure levels in RDD and PDD analyses. Click here for file

Additional File 3 This document provides a table of deviance measures for logistic regression models by tetrachloroethylene exposure levels in RDD and PDD analyses. Click here for file
