# Supplementary material for: Impact of tetrachloroethylene-contaminated drinking water on the risk of breast cancer: Using a dose model to assess exposure in a case-control study
Source: Environ Health. 2005 Feb 25;4:3. doi: 10.1186/1476-069X-4-3 (PMC554766; doi:10.1186/1476-069X-4-3)
Supplement: Additional File 1 — This document describes the dose model in more detail. [file 1476-069X-4-3-S1.doc]

**Dose Model Appendix**

The personal delivered dose model (PDD) sums the contribution of dose from inhalation, ingestion, and dermal absorption for each subject over all their residences. The subject interviews provided information on duration, frequency, temperature of baths and showers, water consumption, and residency time for all residences over a forty-year period. Inhalation doses from baths or showers were calculated as the product of concentration of PCE in the air, inhalation rate, duration, frequency, and residence time, and then summed for an overall inhalation dose. The PCE concentration in air was calculated using the two-resistance theory modeled by Little [11]. The Henry’s law constant was calculated using the temperature-dependent values of vapor pressure, modeled using Antoine’s equation. The overall mass transfer coefficient was calculated as the sum of two resistances in series, from the air film and water film. Similar to models used by McKone [10] and Little [17], the liquid and gas-phase mass transfer coefficients were modeled to be proportional to diffusivity, but the PDD model incorporated temperature-dependent diffusivity and viscosity.

Dose from ingestion was calculated as the product of PCE concentration in the water, the volume of water consumed, and the duration of tap water consumption. No ingestion dose was calculated for years that subjects reported bottled water use. Dose from dermal absorption was calculated using a nonsteady-state application of Fick’s Law developed by the Environmental Protection Agency [12] and based on theoretical work by Cleek and Bunge [18]. The traditional steady-state approach for estimating the dermally absorbed dose of organic chemicals from water was revised by EPA because PCE does not reach steady state during the relatively short contact time of water on a subject’s skin during bathing. The permeability coefficient for PCE was calculated using the equations developed by Potts and Guy [19]. The personal delivered dose model was also developed for trichloroethylene (TCE) and validated using a series of shower experiments by Giardino and Andelman [20]. Sensitivity analyses revealed that the PDD model was most influenced by the initial PCE concentration and residency time and least influenced by temperature [21].
