# Supplementary material for: Impact of tetrachloroethylene-contaminated drinking water on the risk of breast cancer: Using a dose model to assess exposure in a case-control study
Source: Environ Health. 2005 Feb 25;4:3. doi: 10.1186/1476-069X-4-3 (PMC554766; doi:10.1186/1476-069X-4-3)
Supplement: Additional File 2 — This document provides a table of adjusted odds ratios for breast cancer by tetrachloroethylene exposure levels in RDD and PDD analyses. [file 1476-069X-4-3-S2.doc]

Table 4. Adjusted^a^ odds ratios for breast cancer by tetrachloroethylene exposure levels in RDD and PDD analyses.

| Latency period, years | Analysis | Case/  Control (n) | Adjusted ORs  (95% CI) |
| --- | --- | --- | --- |
| 0 |  | | |
| ≤50 Percentile | RDD | 48/44 | 1.0 (0.6-1.6) |
|  | PDD | 41/44 | 0.8 (0.5-1.3) |
| >50 Percentile | RDD | 53/44 | 1.2 (0.8-1.8) |
|  | PDD | 60/44 | 1.3 (0.9-2.0) |
| >75 Percentile | RDD | 32/22 | 1.5 (0.8-2.6) |
|  | PDD | 32/22 | 1.4 (0.8-2.5) |
| >90 Percentile | RDD | 18/9 | 2.1 (0.9-4.8) |
|  | PDD | 10/9 | 1.0 (0.4-2.5) |
| 5 |  | | |
| ≤50 Percentile | RDD | 43/35 | 1.2 (0.7-1.9) |
|  | PDD | 37/35 | 1.0 (0.6-1.7) |
| >50 Percentile | RDD | 44/34 | 1.3 (0.8-2.1) |
|  | PDD | 50/34 | 1.4 (0.9-2.3) |
| >75 Percentile | RDD | 29/17 | 1.7 (0.9-3.2) |
|  | PDD | 29/17 | 1.6 (0.9-3.1) |
| >90 Percentile | RDD | 7/7 | 0.9 (0.3-2.7) |
|  | PDD | 8/7 | 1.0 (0.3-2.8) |
| 7 |  | | |
| ≤50 Percentile | RDD | 34/31 | 1.1 (0.6-1.8) |
|  | PDD | 28/31 | 0.8 (0.5-1.4) |
| >50 Percentile | RDD | 37/30 | 1.2 (0.7-2.0) |
|  | PDD | 43/30 | 1.4 (0.8-2.3) |
| >75 Percentile | RDD | 27/15 | 1.7 (0.9-3.3) |
|  | PDD | 27/15 | 1.7 (0.9-3.4) |
| >90 Percentile | RDD | 7/6 | 0.9 (0.3-2.7) |
|  | PDD | 6/6 | 0.8 (0.3-2.7) |

Table 4 (cont.)

| Latency period, years | Analysis | Case/  Control (n) | Adjusted ORs  (95% CI) |
| --- | --- | --- | --- |
| 9 |  |  |  |
| ≤50 Percentile | RDD | 28/29 | 1.0 (0.5-1.7) |
|  | PDD | 24/29 | 0.8 (0.4-1.4) |
| >50 Percentile | RDD | 35/28 | 1.2 (0.7-2.1) |
|  | PDD | 39/28 | 1.4 (0.8-2.3) |
| >75 Percentile | RDD | 23/14 | 1.7 (0.8-3.4) |
|  | PDD | 22/14 | 1.5 (0.7-3.1) |
| >90 Percentile | RDD | 8/6 | 1.2 (0.4-3.5) |
|  | PDD | 7/6 | 1.0 (0.3-3.1) |
| 11 |  |  |  |
| ≤50 Percentile | RDD | 20/22 | 0.9 (0.5-1.7) |
|  | PDD | 14/22 | 0.6 (0.3-1.2) |
| >50 Percentile | RDD | 29/21 | 1.3 (0.7-2.4) |
|  | PDD | 35/21 | 1.6 (0.9-2.9) |
| >75 Percentile | RDD | 20/11 | 1.8 (0.8-3.8) |
|  | PDD | 21/11 | 1.8 (0.8-3.8) |
| >90 Percentile | RDD | 7/5 | 1.1 (0.3-3.8) |
|  | PDD | 7/5 | 1.2 (0.4-3.9) |
| 13 |  | | |
| ≤50 Percentile | RDD | 19/16 | 1.1 (0.6-2.3) |
|  | PDD | 19/16 | 1.1 (0.6-2.3) |
| >50 Percentile | RDD | 24/16 | 1.5 (0.8-2.9) |
|  | PDD | 24/16 | 1.4 (0.7-2.8) |
| >75 Percentile | RDD | 15/8 | 1.8 (0.7-4.5) |
|  | PDD | 13/8 | 1.4 (0.6-3.6) |
| >90 Percentile | RDD | 5/4 | 1.1 (0.3-4.3) |
|  | PDD | 3/4 | 0.6 (0.1-3.0) |
| 15 |  | | |
| ≤50 Percentile | RDD | 15/11 | 1.4 (0.6-3.0) |
|  | PDD | 18/11 | 1.6 (0.7-3.6) |
| >50 Percentile | RDD | 15/10 | 1.5 (0.7-3.5) |
|  | PDD | 12/10 | 1.1 (0.5-2.8) |
| >75 Percentile | RDD | 12/5 | 2.5 (0.8-7.4) |
|  | PDD | 10/5 | 1.9 (0.6-5.9) |
| >90 Percentile | RDD | 6/2 | -------------^b^ |
|  | PDD | 8/2 | -------------^b^ |

Table 4 (cont.)

| Latency period, years | Analysis | Case/  Control (n) | Adjusted ORs  (95% CI) |
| --- | --- | --- | --- |
| 17 |  | | |
| ≤50 Percentile | RDD | 5/8 | 0.6 (0.2-1.9) |
|  | PDD | 7/8 | 0.9 (0.3-2.5) |
| >50 Percentile | RDD | 10/7 | 1.5 (0.6-4.2) |
|  | PDD | 8/7 | 1.1 (0.4-3.3) |
| >75 Percentile | RDD | 6/4 | 1.6 (0.4-6.2) |
|  | PDD | 6/4 | 1.4 (0.4-5.5) |
| >90 Percentile | RDD | 0/2 | -------------^b^ |
|  | PDD | 1/2 | -------------^b^ |
| 19 |  | | |
| ≤50 Percentile | RDD | 4/3 | 1.4 (0.3-7.0) |
|  | PDD | 5/3 | 1.6 (0.3-7.3) |
| >50 Percentile | RDD | 2/3 | 0.8 (0.1-4.7) |
|  | PDD | 1/3 | 0.4 (0.04-3.5) |
| >75 Percentile | RDD | 1/2 | -------------^b^ |
|  | PDD | 1/2 | -------------^b^ |
| >90 Percentile | RDD | 0/1 | -------------^b^ |
|  | PDD | 0/1 | -------------^b^ |

^a^ The OR was calculated relative to never-exposed cases (n=517) and controls (n=480). Controlled for age at diagnosis or index year, family history of breast cancer, personal history of breast cancer (before current diagnosis or index year), age at first live-birth or still birth, occupational exposure to PCE, and vital status at interview.

^b^ Adjusted analyses were not performed if there were fewer than three exposed cases and three exposed controls.
