# Supplementary material for: Impact of tetrachloroethylene-contaminated drinking water on the risk of breast cancer: Using a dose model to assess exposure in a case-control study
Source: Environ Health. 2005 Feb 25;4:3. doi: 10.1186/1476-069X-4-3 (PMC554766; doi:10.1186/1476-069X-4-3)
Supplement: Additional File 3 — This document provides a table of deviance measures for logistic regression models by tetrachloroethylene exposure levels in RDD and PDD analyses. [file 1476-069X-4-3-S3.doc]

Table 5. Deviance measures for logistic regression models by tetrachloroethylene exposure levels in RDD and PDD analyses.

| Latency period, years | RDD Deviance | | PDD Deviance | |
| --- | --- | --- | --- | --- |
|  | Crude Model | Adjusted Model | Crude Model | Adjusted Model |
| 0 |  | |  | |
| ≤50 Percentile | 1225.3 | 1224.8 | 1202.2 | 1201.6 |
|  | 1225.0 | 1223.8 | 1201.8 | 1200.6 |
| >50 Percentile | 1224.1 | 1222.8 | 1200.7 | 1201.0 |
|  | 1222.9 | 1221.9 | 1199.4 | 1202.2 |
| >75 Percentile | 1225.3 | 1224.8 | 1202.2 | 1201.6 |
|  | 1225.0 | 1223.8 | 1201.8 | 1200.6 |
| >90 Percentile | 1224.1 | 1222.8 | 1200.7 | 1201.0 |
|  | 1222.9 | 1221.9 | 1199.4 | 1202.2 |
| 5 |  | |  | |
| ≤50 Percentile | 1225.0 | 1223.8 | 1201.7 | 1202.2 |
|  | 1224.7 | 1222.7 | 1201.3 | 1199.8 |
| >50 Percentile | 1223.0 | 1225.1 | 1199.4 | 1199.8 |
|  | 1225.3 | 1225.1 | 1202.2 | 1202.2 |
| >75 Percentile | 1225.0 | 1223.8 | 1201.7 | 1202.2 |
|  | 1224.7 | 1222.7 | 1201.3 | 1199.8 |
| >90 Percentile | 1223.0 | 1225.1 | 1199.4 | 1199.8 |
|  | 1225.3 | 1225.1 | 1202.2 | 1202.2 |
| 7 |  | |  | |
| ≤50 Percentile | 1225.3 | 1224.1 | 1202.2 | 1201.8 |
|  | 1225.0 | 1225.3 | 1201.8 | 1200.5 |
| >50 Percentile | 1223.2 | 1225.3 | 1199.7 | 1199.4 |
|  | 1225.3 | 1225.3 | 1202.2 | 1202.1 |
| >75 Percentile | 1225.3 | 1224.1 | 1202.2 | 1201.8 |
|  | 1225.0 | 1225.3 | 1201.8 | 1200.5 |
| >90 Percentile | 1223.2 | 1225.3 | 1199.7 | 1199.4 |
|  | 1225.3 | 1225.3 | 1202.2 | 1202.1 |
| 9 |  | |  | |
| ≤50 Percentile | 1225.1 | 1225.3 | 1202.1 | 1201.4 |
|  | 1225.0 | 1224.3 | 1201.8 | 1200.9 |
| >50 Percentile | 1223.7 | 1224.3 | 1200.3 | 1200.9 |
|  | 1225.2 | 1225.1 | 1202.1 | 1202.2 |
| >75 Percentile | 1225.1 | 1225.3 | 1202.1 | 1201.4 |
|  | 1225.0 | 1224.3 | 1201.8 | 1200.9 |
| >90 Percentile | 1223.7 | 1224.3 | 1200.3 | 1200.9 |
|  | 1225.2 | 1225.1 | 1202.1 | 1202.2 |

Table 5 (cont.)

| Latency period, years | RDD Deviance | | PDD Deviance | |
| --- | --- | --- | --- | --- |
|  | Crude Model | Adjusted Model | Crude Model | Adjusted Model |
| 11 |  | |  | |
| ≤50 Percentile | 1225.0 | 1225.3 | 1201.9 | 1199.7 |
|  | 1224.6 | 1224.2 | 1201.4 | 1199.5 |
| >50 Percentile | 1223.3 | 1224.5 | 1200.2 | 1199.9 |
|  | 1225.1 | 1224.9 | 1202.2 | 1202.2 |
| >75 Percentile | 1225.0 | 1225.3 | 1201.9 | 1199.7 |
|  | 1224.6 | 1224.2 | 1201.4 | 1199.5 |
| >90 Percentile | 1223.3 | 1224.5 | 1200.2 | 1199.9 |
|  | 1225.1 | 1224.9 | 1202.2 | 1202.2 |
| 13 |  | |  | |
| ≤50 Percentile | 1225.3 | 1223.3 | 1202.2 | 1202.1 |
|  | 1224.3 | 1224.1 | 1201.0 | 1201.2 |
| >50 Percentile | 1223.7 | 1225.1 | 1200.5 | 1201.7 |
|  | 1225.3 | 1225.0 | 1202.2 | 1201.9 |
| >75 Percentile | 1225.3 | 1223.3 | 1202.2 | 1202.1 |
|  | 1224.3 | 1224.1 | 1201.0 | 1201.2 |
| >90 Percentile | 1223.7 | 1225.1 | 1200.5 | 1201.7 |
|  | 1225.3 | 1225.0 | 1202.2 | 1201.9 |
| 15 |  | |  | |
| ≤50 Percentile | 1225.0 | 1223.0 | 1201.8 | 1200.7 |
|  | 1224.7 | 1225.3 | 1201.4 | 1202.1 |
| >50 Percentile | 1222.9 | 1224.1 | 1199.4 | 1200.8 |
|  | 1223.5 | 1224.1 | 1200.3 | 1198.4 |
| >75 Percentile | 1225.0 | 1223.0 | 1201.8 | 1200.7 |
|  | 1224.7 | 1225.3 | 1201.4 | 1202.1 |
| >90 Percentile | 1222.9 | 1224.1 | 1199.4 | 1200.8 |
|  | 1223.5 | 1224.1 | 1200.3 | 1198.4 |
| 17 |  | |  | |
| ≤50 Percentile | 1224.3 | 1225.3 | 1201.3 | 1202.1 |
|  | 1225.0 | 1222.7 | 1201.6 | 1202.2 |
| >50 Percentile | 1225.1 | 1225.3 | 1201.8 | 1201.9 |
|  | 1222.4 | 1224.9 | 1199.7 | 1202.0 |
| >75 Percentile | 1224.3 | 1225.3 | 1201.3 | 1202.1 |
|  | 1225.0 | 1222.7 | 1201.6 | 1202.2 |
| >90 Percentile | 1225.1 | 1225.3 | 1201.8 | 1201.9 |
|  | 1222.4 | 1224.9 | 1199.7 | 1202.0 |

Table 5 (cont.)

| Latency period, years | RDD Deviance | | PDD Deviance | |
| --- | --- | --- | --- | --- |
|  | Crude Model | Adjusted Model | Crude Model | Adjusted Model |
| 19 |  | | | |
| ≤50 Percentile | 1225.3 | 1224.8 | 1202.1 | 1201.8 |
|  | 1225.0 | 1222.7 | 1202.0 | 1201.3 |
| >50 Percentile | 1224.9 | 1224.0 | 1202.0 | 1202.0 |
|  | 1223.9 | 1223.9 | 1201.0 | 1201.0 |
| >75 Percentile | 1225.3 | 1224.8 | 1202.1 | 1201.8 |
|  | 1225.0 | 1222.7 | 1202.0 | 1201.3 |
| >90 Percentile | 1224.9 | 1224.0 | 1202.0 | 1202.0 |
|  | 1223.9 | 1223.9 | 1201.0 | 1201.0 |
